# Galactosylated chitosan-functionalized mesoporous silica nanoparticles for efficient colon cancer cell-targeted drug delivery

**DOI:** 10.1098/rsos.181027

**Published:** 2018-12-05

**Authors:** Wei Liu, Yongchao Zhu, Fan Wang, Xue Li, Xiaojing Liu, Jingjing Pang, Weisan Pan

**Affiliations:** 1 Department of Pharmaceutics, School of Pharmacy, Shenyang Pharmaceutical University, 103 Wenhua Road, Shenyang 110016, People's Republic of China; 2 Department of Pharmaceutics, School of Pharmacy, Zhengzhou University, 100 Science Avenue, Zhengzhou 450001, People's Republic of China

**Keywords:** galactosylated chitosan, 5-fluorouracil, mesoporous silica, colon cancer

## Abstract

Targeted drug delivery to colon cancer cells can significantly enhance the therapeutic efficiency. Herein, we developed 5-fluorouracil (5-FU)-loaded amino-functionalized mesoporous silica nanoparticle (MSN-NH_2_)-based galactosylated chitosans (GCs), which are galactose receptor-mediated materials for colon-specific drug delivery systems. Both unmodified and functionalized nanoparticles were characterized by scanning electron microscopy, transmission electron microscopy, X-ray diffraction, Fourier transform infrared spectroscopy, nitrogen sorption and dynamic light scattering. Drug loading capacity and drug release properties were determined by ultraviolet spectrophotometry. 5-FU@MSN-NH_2_/GC showed high loading capacity and possessed much higher cytotoxicity on human colon cancer cells (SW620 cells) than 5-FU@MSN-NH_2_ and free 5-FU. But, MSN-NH_2_/GC did not show significant cytotoxicity. Subsequently, 5-FU@MSN-NH_2_/GC anti-cancer activity on SW620 cells *in vitro* was confirmed by cell apoptosis. These results are consistent with the cellular uptake test in which MSN-NH_2_/GC could specifically recognize and bind to cancer cells by the galectin-receptor recognition. But, it is found that pre-addition of galactose in the medium, leading to competitive binding to the galectin receptor of SW620 cells, resulted in a decrease in the binding of MSN-NH_2_/GC to the galectin receptor. The results demonstrated the inorganic–organic nanocomposite could be used as a promising drug delivery carrier for the targeted delivery of drug into galectin-positive colon cancer cells to improve therapeutic index while reducing side effects.

## 1. Introduction

Colorectal cancer (CRC) is the fourth most frequent cause of cancer-related mortalities in China [1]. The classical clinical treatments for CRC include surgery, radiotherapy and chemotherapy. Of these, chemotherapy is most often used. Among the different chemotherapeutics used to treat CRC, 5-fluorouracil (5-FU), a pyrimidine analogue, which acts as a thymidylate synthase inhibitor, is a first-line anti-cancer drug to combat colon cancer due to its low price and effective anti-cancer activity [2,3]. Usually, 5-FU is administered intravenously, but its clinical application is limited by its unwanted side effects, such as hand and foot syndrome, mucositis/stomatitis, neutropenia, anaemia, nausea/vomiting and cardio-toxicity [4,5]. Various nanocarriers have been developed for encapsulating 5-FU with high loading and minimal side effects. Among them, mesoporous silica nanoparticles (MSNs) have garnered particular attention and have been developed as an ideal host for controlled 5-FU drug delivery [6–11].

Compared with other therapeutic carriers, MSNs have several advantages including low cytotoxicity, high surface areas, low mass density, tunable size and pore diameter that allow fine control of the drug load and the release of kinetics, high adsorption capacity of guest molecules, and high chemical and mechanical stability [12–15]. More importantly, due to the abundant silanol group-containing surface, MSNs are more easily modified with functional groups to allow better control over drug loading and release [10,16].

Functionalized MSNs of various organic groups have been developed for controlled 5-FU release, for example, pyridine-bridged diurea [17], thiol [18], phenyl [15], epidermal growth factor [19,20] and guar gum [8]. But, many of these techniques suffer from lower drug loading capacity and low cell specificity. To overcome this drawback, the present paper is concerned with the galactosylated chitosan (GC) functionalized MSNs.

GC, a derivative of chitosan (CS) which is hydrosoluble at neutral pH, is synthesized by covalently binding d-galactose units to CS through O-1, 6 glycosidic linkages [21,22]. Previous studies suggested that GC has better hydrosolubility, mucoadhesion and cell compatibility compared to CS and maintains low toxicity [23]. Although it has been demonstrated that GC significantly enhanced the hepatocyte-targeting ability compared with CS due to specific ligand receptor interactions between galactose-moieties and asialoglycoprotein receptors [5,24–26], studies on its colon-targeting specificity are limited. Recently, several studies have shown that GC could help to deliver drugs specifically to activated colonic macrophages due to galactose receptor-mediated endocytosis [27,28]. In addition, many studies have shown that galectins, a family of 15 mammalian galactoside-binding proteins, are overexpressed in CRC and play a key role in regulating the development, progression and metastasis of the cancer [29–31]. Moreover, galectins exhibit high affinity for natural small saccharides such as galactose and lactose [31]. To the best of our knowledge, there have been no studies reporting that GC can be used as a carrier for CRC targeting.

The present study aims at developing a targeted drug delivery system based on GC functionalized MSNs, which is able to host high amounts of 5-FU and delivers it in a colon cancer cell-targeted manner.

## 2. Material and methods

### 2.1. Materials

5-FU was purchased from Yuan Cheng Sai Chuang Co., Ltd (China). 3-Aminopropyltriethoxysilane (APTES) was purchased from Sigma-Aldrich (Mainland China). Tetraethylorthosilicate (TEOS) was obtained from Sinopharm Chemical Reagents Co., Ltd (China). CS (molecular weight, 40–60 kDa; deacetylation degree, 91.3%) was purchased from Qingdao Yunzhou Biological Science and Technology Co., Ltd (China). All the chemicals mentioned above were used without further purification. All other regents were of analytical grade. Deionized (DI) water was used throughout the experiments.

### 2.2. Synthesis of mesoporous silica nanoparticles

MSN was prepared using the sol–gel method, as described previously [8,9,11]. In a typical reaction, CTAB (1 g) was dissolved in DI water (480 ml) under stirring and the solution temperature was adjusted to 80°C. Then hydroxide aqueous solution (2.00 M, 3.5 ml) was added to the CTAB solution. After constant stirring for 20 min, TEOS (5 ml) was added drop by drop into the CTAB solution, and the reaction continued for another 2 h. Then, solid crude product was obtained after the reaction mixture was kept for at least 12 h under quiescent conditions. This solid crude product was subsequently centrifuged, washed with DI water and ethanol several times and dried in a vacuum at 50°C overnight to get the unextracted MSNs. MSN (500 mg) was centrifuged after refluxing in an ethanol (50.0 ml)–HCl (0.5 ml, 37.2%) solution at 80°C for 6 h to remove CTAB, and was washed with DI water and ethanol several times and dried in vacuum at 50°C overnight. The obtained product was denoted as MSN.

### 2.3. Synthesis of NH_2_-functionalized MSN (MSN-NH_2_)

Amino-functionalized MSNs were prepared after minor modification of previous works [19,32,33]. Briefly, the prepared MSN was dried at 140°C for 1 h. Then, 1 g of MSN was mixed into 7 ml of APTES. The mixture was added to 50 ml of dry toluene and refluxed at 120°C for 24 h under nitrogen atmosphere. The resulting samples were isolated by centrifugation, repeatedly washed with toluene and methanol and dried in a vacuum at 50°C.

### 2.4. Synthesis of NH_2_-functionalized MSN-based galactosylated chitosan (MSN-NH_2_/GC)

GC (50 mg) was dissolved in the PBS buffer solution (pH = 7.4,10 ml). Then MSN-NH_2_ (20 mg) was suspended into the GC solution and stirred at 25°C for 12 h. The suspension was subsequently centrifuged (13 000 r.p.m., 15 min) and dried at 50°C overnight. The obtained product was denoted as MSN-NH_2_/GC.

### 2.5. Characterizations

Transmission electron microscopy (TEM) images were obtained at 120 kV on a JEM-1200EX transmission electron microscope. TEM samples were obtained by dropping a few drops of an aqueous nanoparticle solution on an ultrathin carbon-supported copper mesh and then evaporating the solvent in an oven at 50°C. Scanning electron microscopy (SEM) micrographs were obtained using a SU8020 field emission SEM at 20 kV and the samples were not electrically conductive and required a gold spray treatment. Particle size and zeta potential of the MSNs, MSN-NH_2_ and MSN-NH_2_/GC were determined by dynamic light scattering (DLS) using a Malvern Zetasizer Nano ZS90 (Malvern Instruments, Worcestershire, UK) at 25°C. MSNs, MSN-NH_2_ and MSN-NH_2_/GC (1 mg ml^−1^) were ultrasonically dispersed in water of pH = 7 (DI water), and the particle size of each sample was measured three times in parallel with disposable polystyrene cells, calculating the average. MSNs, MSN-NH_2_ and MSN-NH_2_/GC (1 mg ml^−1^) were ultrasonically dispersed in water of pH = 7 (DI water) and hydrochloric acid solution of pH = 3 (ion strength of 10–3), each sample was measured in triplicate with a disposable plain folded capillary zeta cell and the average was calculated [34,35].

Small-angle powder X-ray diffraction (XRD) measurements were carried out on an Empyrean Sharp X-ray Diffractometer using CuK*α* radiation (*λ* = 1.54 Å) at 45 kV and 40 mA. The Fourier transform infrared (FTIR) spectra were obtained on an IRAffinity-1 infrared spectrophotometer in the range of 400–4000 cm^−1^ using the KBr pellet technique. Thermogravimetric analysis was carried out on an STA 449 F3 Jupiter from 25 to 900°C with a heating rate of 5°C min^−1^ under nitrogen atmosphere. Nitrogen sorption isotherms were measured on a Micromeritics ASAP 2460 surface area and porosity analyzer at 77 K. The surface area, pore volume and pore size were calculated using the Brunauer–Emmett–Teller (BET) model and Barrett–Joyner–Halenda (BJH) method.

### 2.6. 5-FU loading

5-FU loading into the MSN-NH_2_ was carried out based on the previous method with minor modification [16]. Briefly, MSN-NH_2_ (30 mg) and 5-FU (30 mg) were dispersed in anhydrous ethanol (10 ml). Then the mixture was sonochemically treated for 15 min, using an ultrasonic generator JY92-II with 750 W power. This product was subsequently centrifuged (13 000 rpm, 15 min), and dried in a vacuum at 50°C overnight. The obtained product was denoted as 5-FU@MSN-NH_2_.

GC-capped MSN-NH_2_ (MSN-NH_2_/GC) loaded with 5-FU was carried out by soaking nanoparticles in a concentrated 5-FU solution with stirring. Briefly, CS (2.8 g) and d-galactose (6.0 g) were dissolved in dry Tetrahydrofuran (600 ml), and BF_3_·OEt_2_ (42 ml) was added in the solution with stirring under nitrogen atmosphere. Then the solution was stirred at 60°C under nitrogen atmosphere for 20 h. The crude precipitate was filtered, repeatedly washed with anhydrous methanol and dried in a vacuum at 50°C to get GC [21–23]. GC (50 mg) was dissolved in the PBS buffer solution (pH = 7.4,10 ml) and 5-FU (30 mg) was added in the solution with stirring until 5-FU was solubilized. Then 5-FU@MSN-NH_2_ (20 mg) was suspended into the solution and stirred at 35°C for 12 h. The suspension was subsequently centrifuged (13 000 rpm, 15 min) and dried in a vacuum at 50°C overnight. The obtained product was denoted as 5-FU@MSN-NH_2_/GC.

To evaluate the loading efficiency (%) of 5FU in 5-FU@MSN-NH_2_ or 5-FU@MSN-NH_2_/GC, the supernatants were collected and the free 5-FU concentration was quantitatively analysed using a ultraviolet (UV) spectrophotometer (Shimadzu UV-2550, Japan) at 266 nm.

To measure *in vivo* fluorescence imaging, the fluorescein isothiocyanate (FITC) labelled MSN-NH_2_ (FITC@MSN-NH_2_) and MSN-NH_2_/GC (FITC@MSN-NH_2_/GC) were prepared by minor modification of previous works [19,36], respectively. Twenty micrograms MSN-NH_2_ was dispersed in 10 ml ethanol and mixed with 0.5 ml FITC ethanol solution (1 mg ml^−1^), then stirred for 24 h under dark conditions to obtain FITC-modified MSN-NH_2_ (FITC@MSN-NH_2_). The synthetic process of FITC@MSN-NH_2_/GC was similar to that of 5-FU@MSN-NH_2_/GC.

### 2.7. *In vitro* drug release

The *in vitro* release was studied using a dialysis bag method. Briefly, 5-FU@MSN-NH_2_ and 5-FU@MSN-NH_2_/GC were placed in dialysis membrane bags (MWCO 8–14 kDa), respectively, then immersed in fresh dissolution medium (phosphate-buffered saline (PBS)) (pH = 7.4). The entire system was placed in a shaker incubator set at 60 rpm at 37°C. At predetermined time intervals, 4 ml samples were withdrawn and replaced with equal volume of fresh media to maintain the sink condition. The sample was quantitatively analysed using a UV spectrophotometer (Shimadzu UV-2550, Japan) at 266 nm over a period of 24 h. The percentage of cumulative 5-FU release was calculated and a graph of per cent cumulative release against time was plotted. The studies were conducted in triplicate and the mean results were reported.

### 2.8. Cell culture

Human CRC cell lines SW620 were purchased from Type Culture Collection of the Chinese Academy of Sciences (Shanghai, China) and cultured in Dulbecco's modified eagle medium supplemented with 10% fetal bovine serum, antibiotic (100 units ml^−1^ penicillin and 100 µg ml^−1^ streptomycin) at 37°C in a humidified incubator with 5% CO_2_.

### 2.9. Cellular uptake by fluorescence microscopy

FITC was used as a fluorescent probe and was loaded into MSN-NH_2_ with or without GC to evaluate the cellular uptake qualitatively by a fluorescence microscope. SW620 (containing the receptor of galectins) cells were seeded on 12-well plates (3 × 10^4^ cell/well) and incubated for 24 h. Then, the plates were washed twice with PBS. FITC@MSN-NH_2_ and FITC@MSN-NH_2_/GC (FITC: MSN-NH_2_ or MSN-NH_2_/GC = 1 : 50) were added to the culture medium for 4 h, with cells treated with free FITC in culture medium as a control. The cells were measured using a fluorescence microscope.

For competition assays, the cells were pre-treated with 2 mg ml^−1^ of free galactose before incubation with FITC@MSN-NH_2_/GC. After 30 min incubation, the medium with galactose was aspirated and FITC@MSN-NH_2_/GC was added to the wells at a concentration of 50 µg ml^−1^ for 4 h. The results were compared with FITC@MSN-NH_2_/GC without the addition of galactose [27].

### 2.10. Cellular uptake by flow cytometry

For fluorescent quantitative analysis, SW620 cells were cultured into a 6-well plates (2 × 10^5^cell/well). After 24 h, FITC@MSN-NH_2_/GC (FITC: MSN-NH_2_/GC = 1 : 50) were added to the culture medium. Following incubation at 37°C for 4 h, the cells were washed with PBS three times and harvested by trypsinization. Then the cells were collected by centrifugation and resuspended in PBS for flow cytometric analysis.

To assess whether free galactose concentration has an effect on the cellular uptake, galactose at a final concentration of 2 and 6 mg ml^−1^ was added 30 min prior to FITC-labelled MSN-NH_2_/GC application. After 30 min incubation, the medium with galactose was aspirated and FITC@MSN-NH_2_/GC was added to the wells at a concentration of 50 µg ml^−1^ for 4 h. The results were compared with FITC@MSN-NH_2_/GC in the absence of galactose.

### 2.11. *In vitro* cell viability study

The cell viability of GC, MSN-NH_2_, MSN-NH_2_/GC, 5-FU, 5-FU@MSN-NH_2_, 5-FU@MSN-NH_2_/GC to SW620 cells was assessed by the 3-(4,5-Dimethylthiazol-2-yl)-2,5-diphenyltetrazolium bromide (MTT) assay. The cells were seeded in a 96-well plate (2 × 10^4^cell/well) and incubated for 24 h at 37°C with 5% CO_2_. Afterwards, the culture medium was replaced with a fresh medium containing the GC, MSN-NH_2_, MSN-NH_2_/GC, 5-FU, 5-FU@MSN-NH_2_, 5-FU@MSN-NH_2_/GC at different concentrations and incubated for 48 h, with cells treated with medium only as a control. After being cultured for 48 h, the cells were treated with 20 µl MTT (5 mg ml^−1^ in PBS) for 4 h at 37°C. Then, the medium was removed and 150 µl of dimethyl sulfoxide was added to each well to dissolve the purple formazan. The optical density (OD) of each well was measured at a wavelength of 490 nm by Synergy H1 automatic microplate reader (BioTek, Winooski, VT, USA). The cell viability was calculated.

### 2.12. Apoptosis assay

To study the effect of the sample on apoptosis, the AnnexinV-FITC/propidium iodide apoptosis assay was used to detect SW620 cell apoptosis and death *in vitro*. SW620 cells were seeded in a 6-well plate (3 × 10^5^ cells/well) and cultured for 24 h. Free 5-FU, 5-FU@MSN-NH_2_ and 5-FU@MSN-NH_2_/GC were added to the culture medium at a concentration of 50 µg ml^−1^ and incubated for 24 h. Afterwards, SW620 cells were incubated with 5 ml AnnexinV-FITC and 5 ml propidium iodide for 15 min in the dark. Finally, the samples were measured by flow cytometry.

### 2.13. Mitochondrial membrane potential measurements

SW620 cells were seeded in a 6-well plate (3 × 10^5^ cells per well) and cultured for 24 h. Then, the cells were incubated with media containing samples (5-FU, 5-FU@MSN-NH_2_, 5-FU@MSN-NH_2_/GC) for 24 h. After the incubation, the medium was removed and the cells were then washed with PBS three times. The cells were incubated with 1 ml of the JC-1 (5,50,6,60-tetraethylbenzidazolylcarbocyanine iodide) reagent solution (10 µg ml^−1^) at 37°C for 15 min, washed and analysed using a fluorescence microscope [37,38].

## 3. Results and discussion

**Figure 1.**
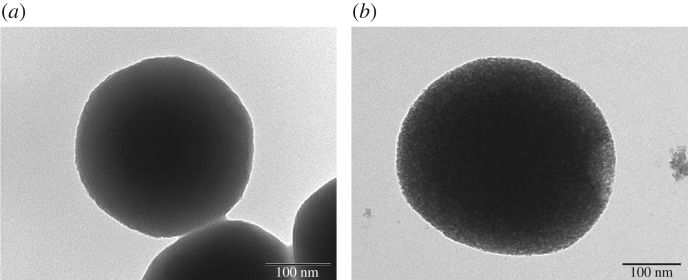
TEM images of MSN (*a*) and MSN-NH_2_/GC (*b*).

**Figure 2.**
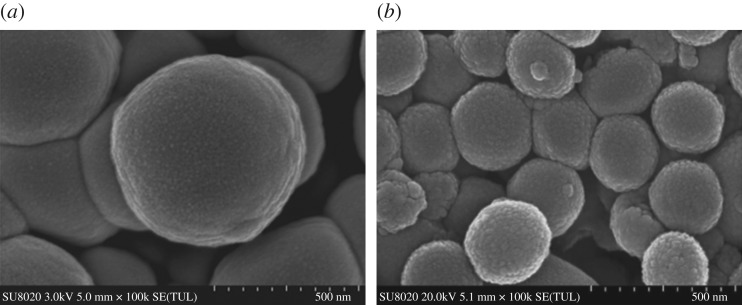
SEM images of MSN (*a*) and MSN-NH_2_/GC (*b*).

**Figure 3.**
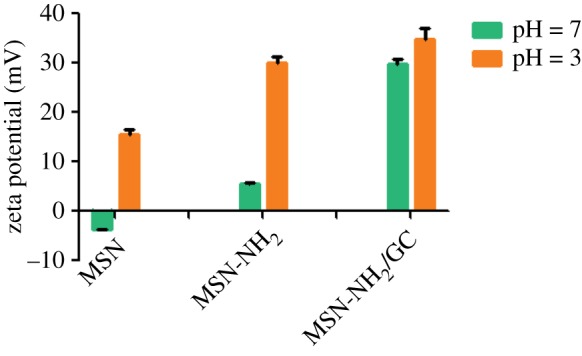
Zeta potential measurements of MSN, MSN-NH_2_ and MSN-NH_2_/GC.

As seen in the TEM images (figure 1*a,b*), MSNs were fully spherical with a size about 250 nm, and there was a clear mesoporous structure with ordered arrangement of mesopores [18]. The TEM images of the MSN-NH_2_/GC, with fuzzy appearance and the lack of visibility of mesoporous channels, indicated a thin layer of GC on the surface of the MSN-NH_2_ as evident in the SEM image shown in figure 2*b*. Compared with MSN (figure 2*a*), the spherical morphology of MSN-NH_2_/GC is not disrupted, but the surface of MSN-NH_2_/GC showed a prominent protrusion and no longer appears smooth, which indicated that MSN-NH_2_ was coated by the GC shell successfully. The average diameter of our optimized MSN formulation is 359.19 ± 9.19 nm; there is little difference between the particle size results measured by TEM (248.59 ± 24.64 nm), the polydispersity index is less than 0.2, which proves good dispersion. We believe that the average nanoparticle size obtained by DLS is larger than the size determined by TEM due to the presence of a hydrated layer around the surface of the particle [39–44]. In addition, the particle size increased to 452.9 ± 4.77 and 511.57 ± 9.69 nm after amino modification and GC encapsulation, respectively. The zeta potential measurements displayed a significant surface charge difference among different formulation nanoparticles (figure 3), presenting further evidence about the presence of coating on the MSN. At pH 7, the zeta potential of MSN nanoparticles was −3.7 ± 0.1 mV, which increased to 5.5 ± 0.3 and 29.9 ± 0.8 mV after amino modification and being caped with GC, respectively. The zeta potential of MSN was negative due to the surface existence of the presence of terminal silanol groups, which were deprotonated at neutral pH [8,45]. After APTES modification, the zeta potential was increased to +5.5 mV due to the positively charged amino groups [9,16]. After being caped with GC, the zeta potential was increased to about 29.9 mV due to C2-NH_2_ group available for protonation [21,23], suggested the successful preparation of MSN-NH_2_/GC. As displayed in figure 3, following pH alteration, the zeta potentials of MSN and MSN-NH_2_ were increased to 15.3 ± 0.96 and 30.0 ± 1.3 mV, respectively, due to the protonation and ionization of silanol groups and NH_2_ groups under acidic conditions.

**Figure 4.**
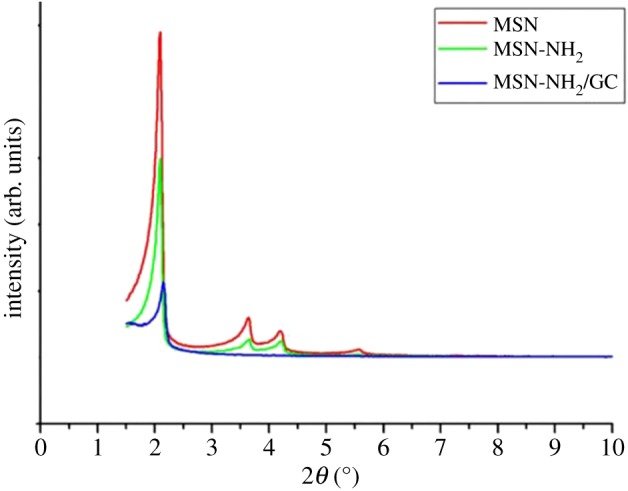
Low angle powder XRD of MSN, MSN-NH_2_ and MSN-NH_2_/GC.

Figure 4 indicates the XRD graphs of MSN, MSN-NH_2_ and MSN-NH_2_ /GC. XRD of MSN shows a very intense diffraction peak at 2*θ* = 2.09 and three weak peaks at 2*θ* values ranging from 3 to 6 that can be indexed as (100), (110), (200) and (210) which indicate two-dimensional ordering and a hexagonal pore structure [15,46–48]. For MSN-NH_2_, three characteristic XRD peaks, (100), (110) and (200), are present which indicate that the ordered mesoporous structure of MSN was retained after modification with amino groups. However, the diffraction intensities decreased, confirming the successful modification by amino groups [49]. MSN-NH_2_/GC has a very weak peak nearly 2, the reflections (110), (200) and (210) are lost. This does not necessary mean the loss of mesoporous ordering, but rather the filling of the mesopores with GC [8,16,46]. The results of SEM (figure 2*b*) and TEM (figure 1*b*) also confirm this.

**Figure 5.**
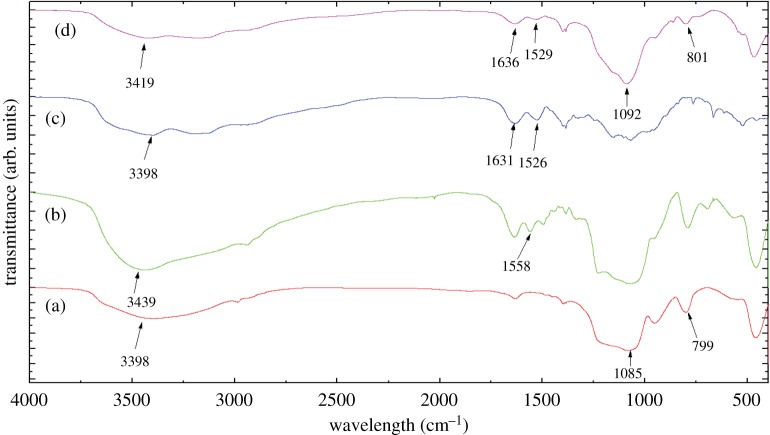
FTIR spectra of MSN (a), MSN-NH_2_ (b), GC (c) and MSN-NH_2_/GC (d).

The FTIR spectra of MSN, MSN-NH_2_ and MSN-NH_2_ /GC are shown in figure 5. The spectrum in figure 5a shows no peaks at 2919, 2890 and 1484 cm^−1^, indicating no template CTAB residue [8]. Figure 5b displays a new absorption band at 1558 cm^−1^ assigned to the N–H asymmetric bending vibration, which confirms the surfaces of MSN were functionalized by the amino groups [12,36,45]. Compared with CS, figure 5c,d shows the peaks of amides I and II of GC appeared at 1633 and 1528, showing bathochromic shift, which confirmed that MSN-NH_2_ was coated by the GC [22,23]. This is consistent with the SEM image (figure 2*b*).

**Figure 6.**
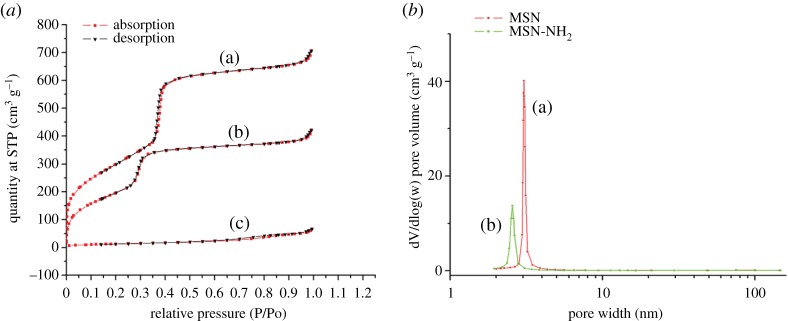
(*a*) Nitrogen adsorption and desorption isotherms for unmodified MSN (a), MSN-NH_2_ (b) and MSN-NH_2_/GC (c). The adsorption branch is black and the desorption branch is red. (*b*) The BJH pore size distributions of MSN (a) and MSN-NH_2_ (b).

Isotherm of nitrogen adsorption/desorption and BJH pore diameters of MSN, MSN-NH_2_ and MSN-NH_2_/GC are represented in figure 6. The MSN and MSN-NH_2_ samples exhibit a type IV isotherm in International Union of Pure and Applied Chemistry (IUPAC) classification, with a sharp inflection positioned at *P*/*P*_0_ around 0.30, which is indicative of the existence of mesoporous structures [7,10,12,48]. The MSN had well-defined mesoporous nanopores with a surface area of 1107.21 m^2^ g^−1^, a pore volume of 1.18 cm^3^ g^−1^ and a BJH diameter of 3.38 nm. After APTES functionalization, the surface area (751.47 m^2^ g^−1^) and the pore volume (0.71 cm^3^ g^−1^) decrease significantly, which demonstrates that the pore channels of MSN were indeed modified by amino groups on their surface. As expected, the GC coating (figure 6) results in a drastic decrease in surface area (47.97 m^2^ g^−1^) and pore volume (0.10 cm^3^ g^−1^), which yielded a flattened isotherm, indicating a significant pore blocking and the subsequent absence of appreciable porosity [8,36,46]. The textural properties of MSN and MSN-NH_2_/GC are represented in figure 1.

The obtained results demonstrated that the 5-FU was encapsulated in amine-functionalized silica nanoparticles with a loading content of 80.6 ± 8.5 mg g^−1^, which is consistent with earlier literature reports [16]. The uptake of 5-FU on functionalized MSNs can take place by means of hydrogen bonding with NH_2_, concomitantly with electrostatic attraction with unfunctionalized silanols, combined with acoustic cavitation aroused under ultrasound action [16,32]. Compared to that of 5-FU@MSN-NH_2_, the loading content of 5-FU@MSN-NH_2_/GC with the GC shell increased 3.1 times, which showed higher loading content than some other new literature for 5-FU immobilization [7,8,50].

**Figure 7.**
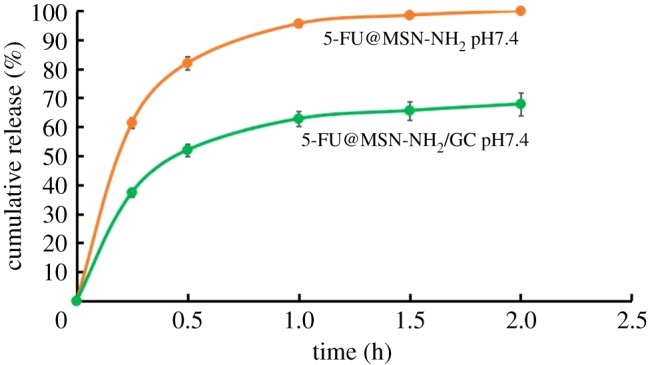
5-FU release profiles from 5-FU@MSN-NH_2_ and 5-FU@MSN-NH_2_/GC in pH solutions (pH = 7.4).

Figure 7 shows the cumulative amount of 5-FU released versus time profiles for 5-FU@MSN-NH_2_ and 5-FU@MSN-NH_2_/GC. In the case of 5-FU@MSN-NH_2_, approximately 80% and 98% of 5-FU was released in pH 7.4 after 0.5 h and 1.5 h, respectively, probably due to the rapid diffusion of 5-FU molecules adsorbed on the surface and its small size (0.5 nm) [10,20]. 5-FU@MSN-NH_2_/GC showed relatively lower drug release, indicating that our use of GC yielded capping.

The release profiles of 5-FU at different pH were mathematically modelled using various kinetic models, including zero-order, first-order, Higuchi, Ritger–Peppas, Baker–Londale and Hixson–Crowell models. The simulation equations and correlation coefficients are calculated and compared in table 1. The best fitting model of 5-FU@MSN-NH_2_/GC is the Korsmeyer–Peppas model. The *n*-value in the Korsmeyer–Peppas model is the release index, and its value depends on the release mechanism. It is reported that *n* < 0.43 corresponds to Fickian diffusion release, and *n*-value between 0.43 and 0.85 represents non-Fickian diffusion release [51,52]. MSN can be considered as a non-expanding spherical sample. The *n*-value of 5-FU@MSN-NH_2_/GC is around 0.25 which is less than 0.43, indicating that the release mode of 5-FU is Fickian diffusion release [11,53,54]. 5-FU@MSN-NH_2_ release data fitted the first-order model better, indicating that the drug was released along the concentration gradient. But, the fitting degree of 5-FU@MSN-NH_2_/GC release data to the first-order diffusion model is not good, which indicated that GC coated on MSN-NH_2_ surface hindered drug release to some extent.

**Table 1.** Release kinetic parameters of 5-FU@MSN-NH_2_/GC and 5-FU@MSN-NH_2._

samples	release medium	release kinetic models	formula	*R*^2^
5-FU@MSN-NH_2_/GC	pH1.2	zero-order	*Q* = 0.2545 + 0.2788*t*	0.6481
		first-order	Ln(1 − *Q*) = −0.3283–0.5238*t*	0.7748
		Higuchi	*Q* = 0.1051 + 0.4869*t*^1/2^	0.8899
		Hixson–Crowell	(1 − *Q*)^1/3^ = 0.8995–0.1398*t*	0.7336
		Korsmeyer–Peppas	InQ = −0.4828 + 0.2545Int	0.9222
		Baker–Londale	3/2[1 − (1 − *Q*)^2/3^]−*Q* = 0.0225 + 0.061*t*	0.8660
5-FU@MSN-NH_2_/GC	pH7.4	zero-order	*Q* = 0.2335 + 0.2775*t*	0.6807
		first-order	Ln(1 − *Q*) = −0.2903–0.5059*t*	0.8011
		Higuchi	*Q* = 0.09 + 0.4784*t*^1/2^	0.9101
		Hixson–Crowell	(1 − *Q*)^1/3^ = 0.9098– 0.1365*t*	0.7624
		Korsmeyer–Peppas	In*Q* = −0.5264 + 0.2824Int	0.9341
		Baker–Londale	3/2[1 − (1 − *Q*)^2/3^]−*Q* = 0.0179 + 0.0578*t*	0.8920
5-FU@MSN-NH_2_	pH1.2	zero-order	*Q* = 0.3689 + 0.4004*t*	0.6439
		first-order	Ln(1 − *Q*) = −0.1616–2.6464*t*	0.9940
		Higuchi	*Q* = 0.1534 + 0.7005 ×*t*^1/2^	0.8873
		Hixson–Crowell	(1 − Q)^1/3^ = 0.8927–0.4541*t*	0.9607
		Korsmeyer–Peppas	In*Q* = −0.1162 + 0.2514Int	0.9222
		Baker–Londale	3/2[1 − (1 − *Q*)^2/3^]−*Q* = 0.0344 + 0.2503*t*	0.9685
5-FU@MSN-NH_2_	pH7.4	zero-order	*Q* = 0.3849 + 0.3936*t*	0.6187
		first-order	Ln(1 − *Q*) = −0.1943–2.7781*t*	0.9903
		Higuchi	*Q* = 0.1672 + 0.6959 *t*^1/2^	0.8702
		Hixson–Crowell	(1 − *Q*)^1/3^=0.8778–0.4525*t*	0.9497
		Korsmeyer–Peppas	In*Q* = −0.1036 + 0.2313Int	0.9107
		Baker–Londale	3/2[1 − (1 − *Q*)^2/3^]−*Q* = 0.0444 + 0.2491*t*	0.9522

**Figure 8.**
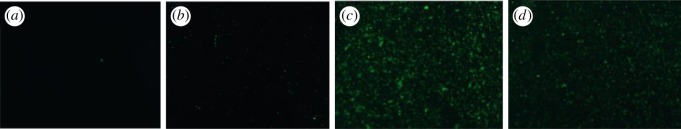
The fluorescence microscopy photographs of SW620 cells internalizing the FITC (*a*), FITC@MSN-NH_2_ (*b*), FITC@MSN-NH_2_/GC (*c*), FITC@MSN-NH_2_/GC+galactose (*d*).

To evaluate the *in vitro* cancer targeting ability of the carrier, we compared the cellular uptake efficiencies of MSN-NH_2_, MSN-NH_2_/GC and MSN-NH_2_/GC+ galactose by using a fluorescence microscope. As shown in figure 8, the green fluorescence intensity of the FITC@MSN-NH_2_/GC group is significantly higher than FITC@MSN-NH_2_, revealing the high specificity of MSN-NH_2_/GC to galectins-positive cells. But, it is found that pre-addition of galactose (adding 2 mg ml^−1^ galactose to medium 30 min in advance) in the medium, with competitive binding to the galectins receptor of SW620 cells, resulted in a decrease in the binding of MSN-NH_2_/GC to the galectins receptor. Meanwhile, the green fluorescence of MSN-NH_2_/GC with free galactose group was more apparent than that of the MSN-NH_2_ group with positive charges providing the specific electrostatic affinity to the cell membrane [55], which indicates that free galactose as a competitive inhibitor competes with MSN-NH_2_/GC and inhibits their cellular uptake in SW620 cells, but free galactose may not be able to totally block the galactose-receptor recognition on the cell surface [19].

**Figure 9.**
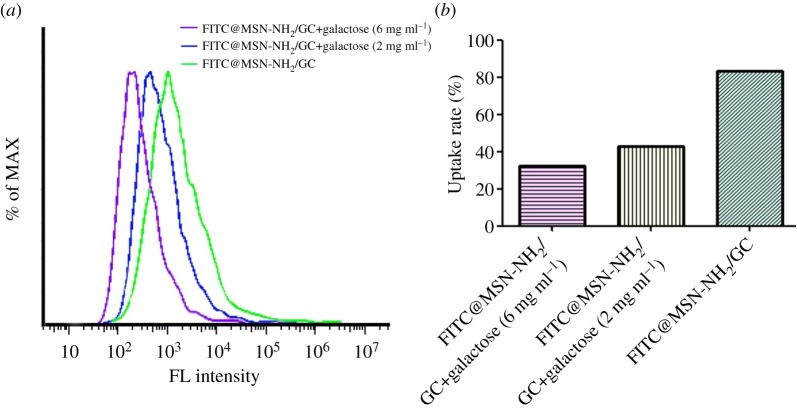
The fluorescence intensity of the samples in SW620 cells (*a*). The uptake rates of the samples in SW620 cells (*b*).

The internalized fraction of the samples taken up by the SW620 cells was evaluated by flow cytometry and the results are shown in figure 9. The uptake rates of the MSN-NH_2_/GC + galactose (6 mg ml^−1^), MSN-NH_2_/GC + galactose (2 mg ml^−1^), MSN-NH_2_/GC are 32.1%, 42.8% and 83.2%, respectively. The uptake of MSN-NH_2_/GC is much higher than that of MSN-NH_2_/GC as galactose were added in advance in SW620 cells and the uptake rate of MSN-NH_2_/GC + galactose (6 mg ml^−1^) group was lower than that of MSN-NH_2_/GC@galactose (2 mg ml^−1^) group. The higher the concentration of galactose added in the medium in advance, the faster the galactose binds to the galactose receptor of SW620 cells, resulting in a decrease in the binding of MSN-NH_2_/GC to the galactose receptor, so the MSN-NH_2_/GC + galactose uptake rate was lower than that of the MSN-NH_2_/GC group. The experimental results show that MSN-NH_2_/GC could specifically recognize and bind to galectins-positive cancer cells by the galactose-receptor recognition, which is consistent with the findings in cellular uptake by fluorescence microscopy.

**Figure 10.**
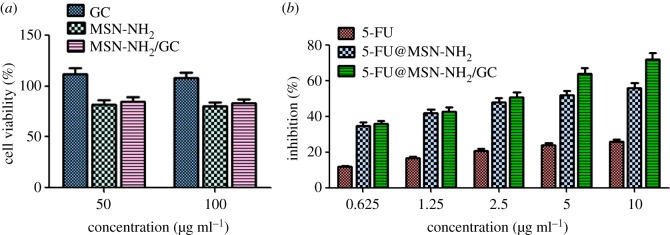
Cell viability assay of GC, MSN-NH_2_ and MSN-NH_2_/GC on SW620 cells at different concentrations (*a*). Cell inhibition rate of free 5-FU, 5-FU@MSN-NH_2_ and 5-FU@MSN-NH_2_/GC on SW620 cells at different concentrations (*b*).

The viability of SW620 cells treated with GC, MSN-NH_2_ and MSN-NH_2_/GC was analysed by MTT assay for 48 h. As shown in figure 10*a*, when GC, MSN-NH_2_ and MSN-NH_2_/GC were at concentrations of 50 and 100 µg ml^−1^, the viability of the treated groups was also above 80%. The results showed that GC, MSN-NH_2_, MSN-NH_2_/GC nanocarriers had no apparent cytotoxicity on SW620 cells [56], indicating their biocompatibility.

We further sought to detect the presence of any drug toxicity at different concentrations (0.625, 1.25, 2.5, 5, 10 µg ml^−1^) by the MTT assay. The cell viability of free 5-FU, 5-FU@MSN-NH_2_, 5-FU@MSN-NH_2_/GC on SW620 cells is shown in figure 10*b*. Both 5-FU@MSN-NH_2_/GC and 5-FU@MSN-NH_2_ were significantly more cytotoxic to SW620 cells than free 5-FU, and 5-FU@MSN-NH_2_/GC exhibited higher cytotoxicity than 5-FU@MSN-NH_2_ at all treated concentrations. For instance, 5-FU@MSN-NH2/GC and 5-FU@MSN-NH_2_ with a 5-FU concentration of 5 µg ml^−1^ can achieve nearly 64% and 52% of cancer cell growth inhibition, while this inhibition increased to about 72.5% and 56% when the particle concentration increased to 10 µg ml^−1^ for 5-FU@MSN-NH_2_/GC and 5-FU@MSN-NH_2_, respectively. Compared with free 5-FU, cell cytotoxicity of 5-FU@MSN-NH_2_/GC increased by 3.2 times at the same concentration of 10 µg ml^−1^. It may be related to the fact that galactose in 5-FU@MSN-NH_2_/GC binds to the galactose receptor of SW620 cells and increases cellular uptake of drugs [27]. With the specific cellular uptake of 5-FU@MSN-NH_2_/GC in SW620 cells, more 5-FU can be delivered to cells to cause cell death and the sensitivity of cells to 5-FU can be restored. This is consistent with the flow cytometry results shown in figure 9 in which GC grafting on the MSN-NH_2_ surface can significantly facilitate the cellular uptake in galectin-expressed cancer cells.

**Figure 11.**
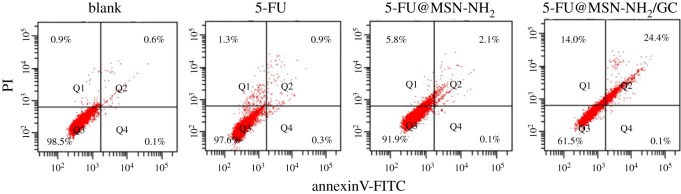
Flow cytometry measurement of SW620 cells apoptosis treated with 5-FU, 5-FU@MSN-NH_2_ and 5-FU@MSN-NH_2_/GC.

As shown in figure 11, the cell apoptosis analysis of free 5-FU, 5-FU@MSN-NH_2_ and 5-FU@MSN-NH_2_/GC was characterized by counting the numbers of viable cells, necrotic cells, early apoptosis cells and late apoptosis cells, respectively. Compared with the control, free 5-FU, 5-FU@MSN-NH_2_ and 5-FU@MSN-NH_2_/GC could significantly cause the late apoptosis or necrosis but no significant effects on early apoptosis on SW620 cells because 5-FU affects the cell physiology in the S-phase (synthesis phase) [10]. The late apoptotic cell populations were 0.9%, 2.1% and 24.4% for free 5-FU, 5-FU@MSN-NH_2_ and 5-FU@MSN-NH_2_/GC, respectively. The induced necrosis percentages by free 5-FU, 5-FU@MSN-NH_2_ and 5-FU@MSN-NH_2_/GC were 1.3, 5.8 and 14.0, respectively. This phenomenon may be caused by MSN-NH_2_/GC increase in cellular uptake of drugs [56]. With the specific cellular uptake of 5-FU@MSN-NH_2_/GC in SW620 cells, more 5-FU can be delivered to cells to cause cell apoptosis or necrosis. Under the same concentration, compared with the untargeted drug delivery system, the targeted drug delivery system can be more effective on cell apoptosis.

**Figure 12.**
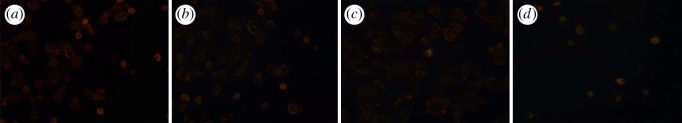
Mitochondrial membrane potential measurements of blank (*a*) free 5-FU, (*b*) 5-FU@MSN-NH_2_, (*c*) 5-FU@MSN-NH_2_/GC (*d*) on SW620 cells.

JC-1 is a highly specific dye that selectively enters the mitochondria of cells and reversibly changes its fluorescence from red to green as the mitochondrial membrane potential decreases [38,57]. It could be seen that from figure 12 that green is more and more obvious, which shows 5-FU@MSN-NH_2_/GC can significantly increase the proportion of green fluorescence in cancer cells and induce tumour cell death by depolarizated mitochondrial membrane.

## 4. Conclusion

In this study, the GC, which was grafted galactose onto CS to produce target-cell specificity and improve the solubility, was successfully cavered to the surface of MSN-NH_2_ based on nanoparticle characterization to deliver 5-fluorouracil. The GC caped increased the zeta potential and showed high loading capacity of 5-FU. 5-FU@MSN-NH_2_/GC demonstrated a sustained release behaviour than 5-FU@MSN-NH_2_. The galactose receptor of MSN-NH_2_/GC was able to recognize and specifically bind to the galectin receptor in SW620 cells, confirmed by fluorescence microscopy and flow cytometry. As a result, these smart nanoparticles also demonstrate effectiveness in enhancing the anti-cancer activity of chemotherapeutic drugs towards SW620 cells through *in vitro* cytotoxicity, cell apoptosis analysis and mitochondrial membrane potential measurements. These results suggested that MSN-NH_2_/GC may potentially be used as a promising drug delivery carrier for the targeted delivery of a drug into galectin-positive colon cancer cells.

## Data accessibility

This article does not contain any additional data.
